# Effect of roadside trees on pedestrians’ psychological evaluation of traffic noise

**DOI:** 10.3389/fpsyg.2023.1166318

**Published:** 2023-08-16

**Authors:** Thulan Nguyen, Makoto Morinaga

**Affiliations:** ^1^Department of Architectural Design, Interdisciplinary Faculty of Science and Engineering, Shimane University, Matsue, Shimane, Japan; ^2^Department of Architecture and Building Engineering, Faculty of Architecture and Building Engineering, Kanagawa University, Yokohama, Kanagawa, Japan

**Keywords:** preference, roadside tree, audio-visual stimuli, psychological evaluation, soundscape, noise control, binaural recordings, environmental comfort

## Abstract

**Introduction:**

This study aims to investigate the interplay between roadside trees and pedestrians’ assessment of traffic noise and comfort. The study examines the potential effects of visual and design elements of roadside trees on the overall soundscape comfort.

**Methods:**

The study design involves a systematic exploration of different conditions, encompassing traffic volume, distance from sound source, and tree density. For each combination, two experimental scenarios are created: (1) participants experience a binaural sound recording exclusively, and (2) participants experience the same binaural recording while concurrently immersed in a virtual reality (VR) video.

**Results:**

Analysis of participants’ noise perception, measured using a quiet-noisy scale, reveals no significant disparity between conditions. This suggests that the mere presence of roadside trees does not necessarily lead to a perceived reduction in noise loudness. However, evaluation of sound intensity exposes a notable discrepancy between low and medium tree density levels. Furthermore, the study confirms the impact of roadside tree visibility, with scenes containing trees yielding more positive evaluations compared to sound-only scenarios. Remarkably, the absence of trees in the roadside scene garners consistent evaluations across both experimental conditions. Significantly, higher roadside tree density in conjunction with the combined sound and VR video condition prompts a more favorable assessment than the sound-only scenario.

**Discussion:**

While the study indicates that roadside trees might not substantially mitigate perceived physical noise levels, their influence on the psychological well-being of urban inhabitants is considerable. The findings highlight that even though these trees may not overtly diminish noise, they hold substantial potential to enhance the overall comfort and well-being of city residents. This underscores the multifaceted benefits of integrating green spaces into urban design for improving the quality of urban soundscapes and residents’ experiences.

## Introduction

Many regulations, guidelines, and policies are intended to mitigate the negative effects of environmental noise on urban residents (Berglund et al., 1999; International Organization for Standardization, 2003; World Health Organization, 2018). A multitude of solutions for reducing noise have been proposed, taking into account that noise is reduced by energy attenuation due to distance and air absorption. However, soundscape design takes a different approach, departing from the traditional level-based method that treats sound as a waste product to be reduced and managed (Brown, 2011). The soundscape field views sound as a resource, like other scarce resources, and seeks to protect and enhance it where appropriate (Brown, 2015). Recent studies in acoustic comfort have shown that reducing sound levels does not necessarily lead to a better sound environment in urban areas (Yang and Wang, 2005; Lavandier and Defreville, 2006; Axelsson et al., 2010; Samara and Tsitsoni, 2011). The focus of recent soundscape research is on sounds of preference and the users’ perception of sound, aiming to improve acoustic comfort. Furthermore, given that soundscape preference is greatly influenced by local needs, cultural aspects, and the landscape, it is crucial to broaden the study of soundscape in various social and cultural environments and natural conditions.

For a long time, plants have been preferred elements in the city landscape due to their esthetic appeal and air-purifying capabilities. Along roadsides, trees are planted to enhance visual quality and restore the natural environment. Roadside trees are also expected to have a positive effect on pedestrian psychological health, through visual effects and physical sound reduction. Yang et al. investigated psychological benefits provided by urban parks and landscape environments (Yang et al., 2011). The study provides evidence that landscape plants in urban parks and other green environments have psychological benefits in terms of attenuating noise and influencing individuals’ emotional responses. A field experiment in which participants were exposed to different urban environments, including a park, a street, and a built-up area, found that participants who were exposed to the park environment experienced greater stress relief compared to those who were exposed to the street or built-up area (Tyrväinen et al., 2014). Kardan et al. found that living near green spaces in a large urban center is associated with better self-reported health and lower levels of perceived stress (Kardan et al., 2015). Roe et al. found that exposure to green space reduces cortisol levels, which are a physiological marker of stress, in deprived urban communities (Roe et al., 2013). All these studies suggest that green spaces have the potential to improve mental health and wellbeing in urban areas.

It is essential to understand the sound perception of urban residents and its association with people’s well-being in the context of urban green. Although previous studies have shown positive associations between exposure to green spaces and acoustic comfort and overall well-being indicators, there is a lack of research that quantifies the psychological impact on the population. Specifically, no existing studies have specifically addressed this impact of roadside tree in relation to psychological sound indices such as loudness and sharpness. In this study, our aim is to bridge these gaps by taking a multidisciplinary approach to understanding the sound perception of urban residents in relation to urban green environments.

Furthermore, in order to assess the methods and techniques used to evaluate the acoustic environment, we will also compare subjective evaluations considering both audio and visual aspects of the soundscape. Currently, discussions have taken place on various aspects of soundscape evaluation and the utilization of audio and audiovisual scene analysis techniques to assess the sound (Kang and Schulte-Fortkamp, 2016; Aletta and Astolfi, 2018; Hasegawa and Lau, 2021; Thompson, 2021). However, it remains to be determined whether subjective evaluations of noise reduction effectiveness vary depending on whether audio or audiovisual scenes are utilized. By distinguishing the pedestrians’ psychological evaluation of traffic noise in various exposure scenes, our aim is to assess the importance of visual stimuli in shaping individuals’ experience of the same sound environment.

We will examine the relationship between the physical noise reduction effect and psychological evaluation, specifically focusing on how roadside trees affect pedestrians’ psychological evaluation of traffic noise and whether they have a noise reduction effect. Through investigating the connection between the psychological-physical noise reduction effect and the design of roadside trees, this study will provide valuable references for designing urban green spaces as an effective noise mitigation measure. Our findings are expected to be beneficial for municipal governments, environmental managers, landscape planners, and policymakers in their efforts to create more comfortable spaces for communities.

## Methods

### Study sites

**Figure 1:**
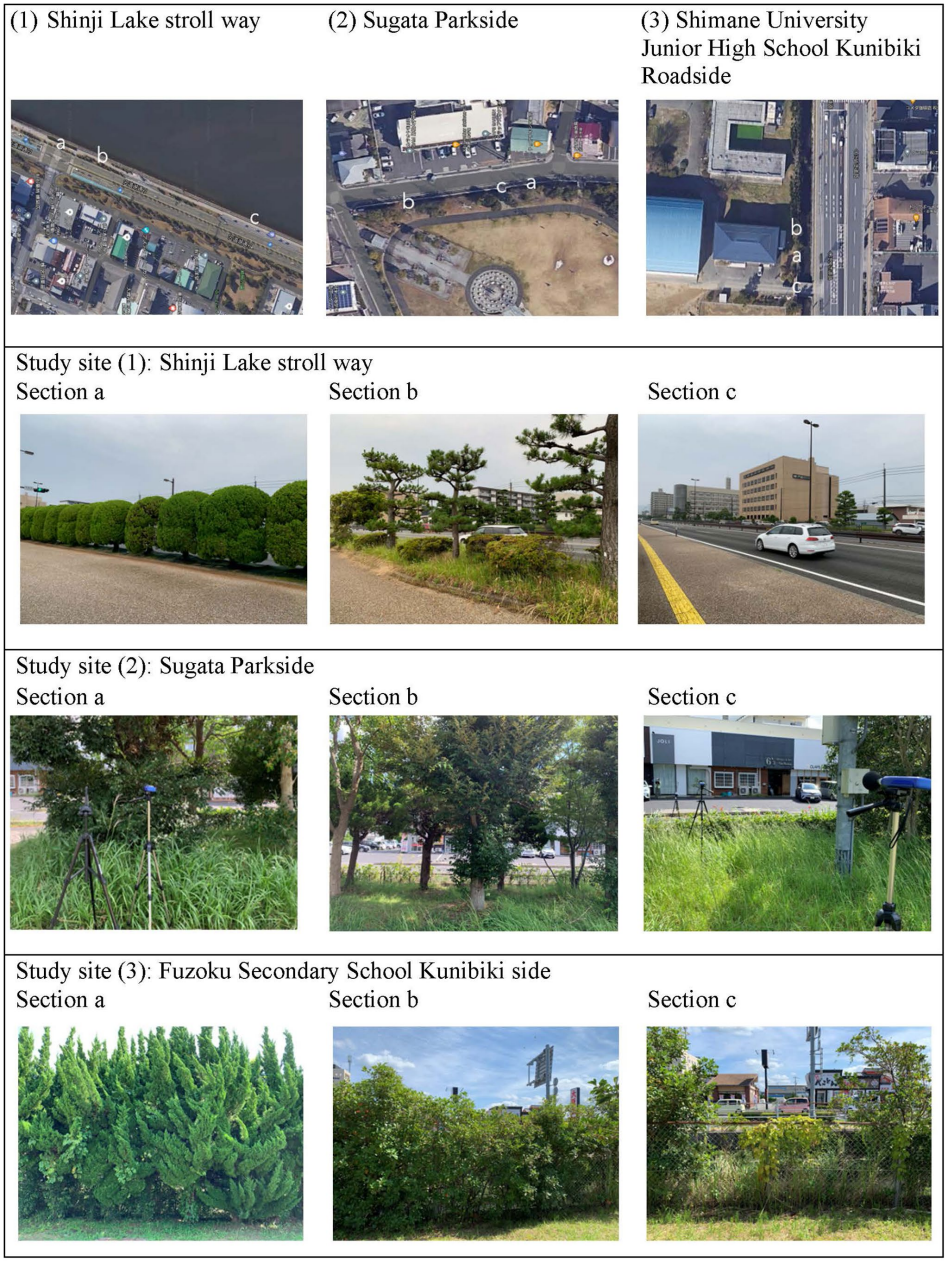
Satellite image of the three study sites and measurement sections at each study site.

Three sites were selected for this study based on their different landscape and acoustical characteristics. The study sites were located along different roads in Matsue City, taking into account varying traffic volumes and vehicle speeds. Figure 1 presents images of the study sites and measurement sections at each study site.

Study site No. 1, the Shinji Lake Stroll Way, is a park promenade that runs around Shinji Lake. One side of the promenade faces the water surface, while the other side faces an inter-provincial four lanes road with heavy truck traffic and average vehicle speed of about 60 km/h.

Study site No. 2, the Sugata Parkside, is a walkway in a park located in a residential area. The two-lanes road that runs by the park is narrow and very little traffic, mainly small cars and pedestrians, and average vehicle speed of about 30 km/h.

Study site No. 3, the Shimane University Junior High School Kunibiki Roadside, is the campus of the junior high school located near a traffic intersection with signal lights. The road has a large traffic volume and the flow of vehicles moves slowly at average vehicle speed of about 30 km/h. The campus is located on a small canal and a wide sidewalk away from the main inner-city road.

Previous studies (Jang et al., 2015; Michta and Haniszewski, 2018; SAE International, 2020) have suggested that study sites should be selected based on the relationship between traffic volume, vehicle speed, and noise levels. This takes into account the different noise levels generated by varying levels of traffic volume and vehicle speed on the roads. As shown in Figure 1, the impact of roadside trees was assessed at each study site in three sections, chosen based on tree density from high, medium, to low or none; for instance, at study site No. 1 (Shinji Lake Stroll Way), section a is separated from the driveway by a row of tall poplar trees, section b has a line of sparse trees mixed with shrubs and tall trees, and section c has no trees.

### Physical index data

**Figure 2:**
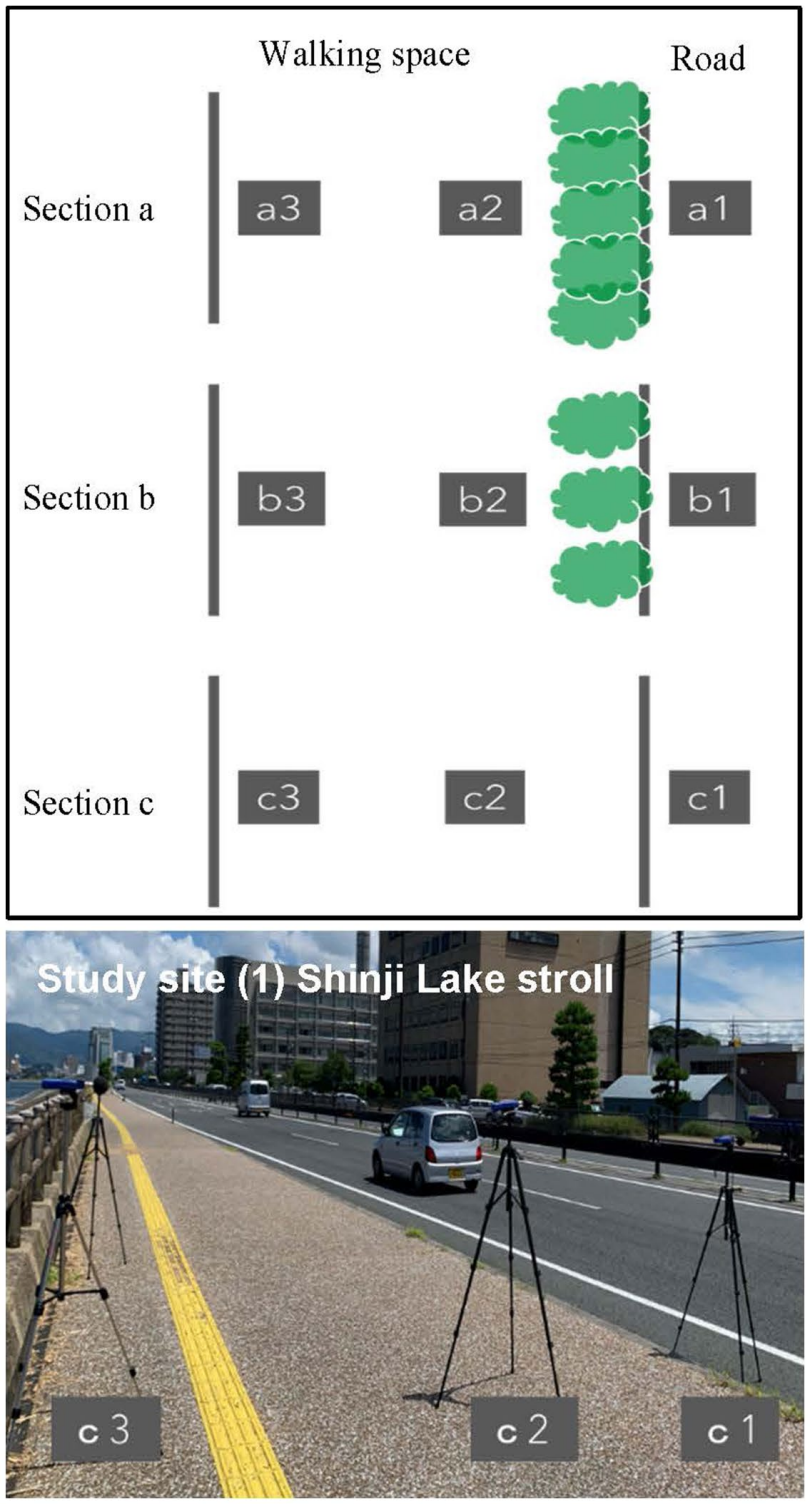
Measurement location at each site defined by the distance from the road axis and an example of measurement locations c1-3 at the study site (1) shinji Lake stroll way.

Physical index data was collected to investigate the noise-reducing effect of roadside trees. Figure 1 depicts the measurement contexts in three sections representing different levels of vegetation density for each study site. Section a represents a situation of dense vegetation with no road visible. Section b represents a situation of medium vegetation density with the road visible. Section c represents a situation of little or no vegetation. At each section, A-weighted sound pressure level was measured for 10 min at three distances from the road (Figure 2). Three sound level meters (RION NL-42) were positioned at a height of 1.5 m to measure the exposure level experienced by a standing person. The microphones were placed at least 2 m away from any reflective surfaces to minimize the influence of early reflections and echoes on the recorded sound. This distance ensures a cleaner and more accurate representation of the soundscapes (Morillas et al., 2016; International Organization for Standardization, 2017).

Figure 2 shows the measurement settings at three locations at Study Site 1 (Shinji Lake stroll way). Location 1 is along the road, location 2 is behind the trees on the roadside (walking space), and location 3 is on the sidewalk (walking space), further away from the trees than location 2. Binaural soundscape clips were sampled at each measurement location for 20 s using the HEAD acoustics B2U and BHS I headphone. 360° video data was collected at each location using the RICOH THETA V camera to reproduce the study area’s environment in virtual audio-visual subjective experiments. The choice of a shorter 20-s sample duration for the binaural and video recording was influenced by the time constraints that participants would have for completing the subjective evaluation within approximately 1 h. This shorter sample duration was selected to strike a balance between participants’ concentration during the tests and the need to capture relevant acoustic features or variations within the study context.

### Subjective evaluation experiments

Twenty students aged 20 to 25 participated in the experiment. All the participants had normal hearing and vision, and they were informed about the experiment’s aim and protocol. Two stimuli scenes were created by combining binaural recordings and video data. The subjects evaluated two stimuli scenes: (1) listening to the binaural recording sound only, and (2) listening to the binaural recording sound while watching a VR video. The second stimuli scene was designed to examine whether the presence or absence of roadside trees affected people’s noise evaluation. A calibration procedure was implemented to ensure the consistency of playback levels for the headphones used in the listening tests, relative to the sound pressure levels experienced during field recording. The laboratory environment was controlled uniformly under three conditions: a background noise level of less than 30 dB, a room temperature of 25°, and work surface illumination of 110 lux.

As described in section Physical index data, binaural soundscape clips and 360° video data were sampled at three locations in each of the three sections in all study sites. A total of 27 samples were collected to reproduce the audio-virtual environment of the study area. Twelve of these samples were used in the subjective evaluation experiment, as shown in Table 1. The virtual reality (VR) equipment, Oculus Quest 2, was used to present a complete and realistic visual environment of the walking space, with different situations involving a roadside tree.

**Table 1:** Samples used for subjective evaluation experiment.

Features	Study sites and locations name
Areas alongside the road	Shinji Lake stroll way a1
	Sugata Parkside a1
	Fuzoku Secondary School Kunibiki side a1
Areas far from the road and having a dense plant	Shinji Lake stroll way a3
	Sugata Parkside a3
	Fuzoku Secondary School Kunibiki side a3
Areas far from the road and having a medium-dense plant	Shinji Lake stroll way b3
	Sugata Parkside b3
	Fuzoku Secondary School Kunibiki side b3
Areas far from the road and having little or no plant	Shinji Lake stroll way c3
	Sugata Parkside c3
	Fuzoku Secondary School Kunibiki side c3

A questionnaire was developed to assess the evoked state associated with experiencing sound and audio-visual stimuli. Acoustic comfort is the most widely used perceptual attribute in relevant research for evaluating the impact of the acoustic environment and the soundscape quality (Zhang et al., 2018). In this study, nine perceptual attributes, including preference, pleasantness, naturalness, powerfulness, speed, quietness, safety, relaxation, anxiety, and stressfulness, were used to measure the psychological responses to traffic noise and a roadside soundscape. To ensure the reliability and validity of the questionnaire, before implementing it in the study, preliminary experiments was conducted to refine the questionnaire and gather feedback from participants on its clarity and relevance.

**Figure 3:**
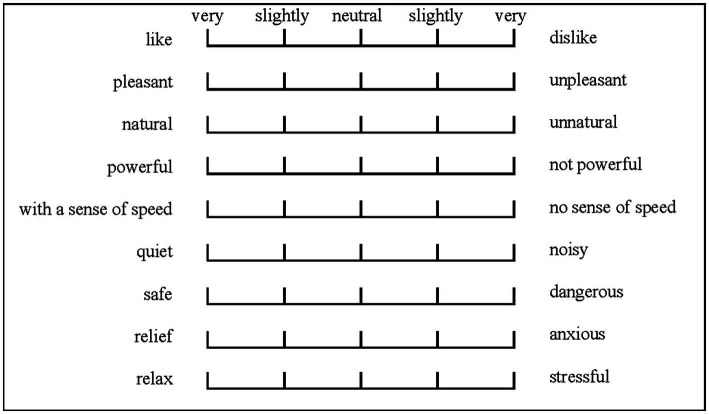
Format of an answer sheet of a subjective evaluation experiment.

As shown in Figure 3, the evaluation sheet used in the subjective evaluation experiment consisted of a five-point verbal scale with the following verbal marks: (1) “like/dislike”; (2) “pleasant/unpleasant”; (3) “natural/unnatural”; (4) “powerful/not powerful”; (5) “with a sense of speed/no sense of speed”; (6) “quiet/noisy”; (7) “safe/dangerous”; (8) “relaxing/anxiety-provoking”; and (9) “relaxing/stressful.” As suggested by Likert (1932), a five-point verbal scale was used in this study for self-assessment, with five answer options containing two extreme poles, “very,” and a less intense option, “slightly,” connected by a neutral answer option, “neutral.” The evaluation words in the questionnaire were presented in the same order for all experiments. Participants first rated their preference for the samples, such as “like” or “pleasant,” then rated other items relating to the features of the samples, such as “natural,” “powerful,” and “sense of speed,” and finally, items that reflect the influence of traffic noise.

### Data analysis

The measured values of sound level meters and binaural recordings were analyzed to find the acoustic characteristics of the sound environment at each location. Artemis SUITE 12.0 was used to analyze the captured materials and generate psychoacoustic indicators such as sharpness and loudness. The density of vegetation and distance from the road could alter the frequency characteristics of road traffic noise, potentially affecting loudness and sharpness. However, roughness and fluctuation strength, which are time-varying characteristics, are not expected to undergo significant changes. Therefore, this study does not address roughness and fluctuation strength as specific parameters of investigation. Sharpness is the ratio of the loudness of high-frequency sounds to the overall loudness of a sound, representing the “center of gravity” of the frequency spectrum scene and does not depend on the sound pressure level. The higher the center of gravity, the sharper the sound. The correlation between measured physical and psychological indexes will provide insight into the psychological noise reduction effect of roadside trees.

The measured values of physical indicators, such as sound pressure level (dB), sharpness (acum), and loudness (sone), at locations with higher vegetation density were compared to those in less dense areas. For example, the discrepancy in the level difference between a1 and a3 and between c1 and c3 will clarify the noise reduction effect of roadside trees. The distance reduction equation (Maekawa, 1970) was used to distinguish the distance reduction effect from that caused by vegetation. The sound attenuation formula is as follows:


(1) $${\mathrm{SPL}}_{2}={\mathrm{SPL}}_{1}-20\log\left(R_{1}/R_{2}\right)$$

where: SPL_1_: Sound pressure level at point 1;

- SPL_2_: Sound pressure level at point 2;
- R_1_: Distance from the sound source to point 1; and
- R_2_: Distance from the sound source to point 2.

The association between the psychological assessment data from the subjective evaluation experiment, the measured values, and the evaluations by multiple subjects was analyzed.

## Results

### Effect of physical noise reduction

Table 2 presents the equivalent continuous A-weighted sound pressure levels (L_Aeq_, dB) measured at all study sites. The L_Aeq_ values at locations a1, b2, and c1 exhibited variations across the study sites, which can be attributed to differences in traffic volumes and vehicle speeds. Specifically, at section a of the Shinji Lake Stroll Way, the L_Aeq_ values ranged from 62.0 dB to 73.8 dB. To provide a contextual understanding of these noise levels, it is worth noting that this range is comparable to sound samples produced by passenger cars (50–65 dB) and trucks (65–75 dB; U.S. Department of Transportation Federal Highway Administration, 2011).

**Table 2:** Equivalent continuous A-weighted sound pressure levels (L_Aeq_, dB) at all study sites.

Plant density condition	Measurement locations	Shinji Lake stroll way	Sugata Parkside	Fuzoku School Kunibiki side
High-density plants	a1	69.7	57.4	65.0
	a2	66.2	54.2	56.7
	a3	62.0	51.8	55.3
Low-density plants	b1	73.8	59.2	65.3
	b2	69.0	55.3	59.8
	b3	66.1	57.2	59.3
Very little or no plants	c1	72.3	55.0	72.3
	c2	70.7	51.8	67.8
	c3	68.8	50.1	66.0

**Figure 4:**
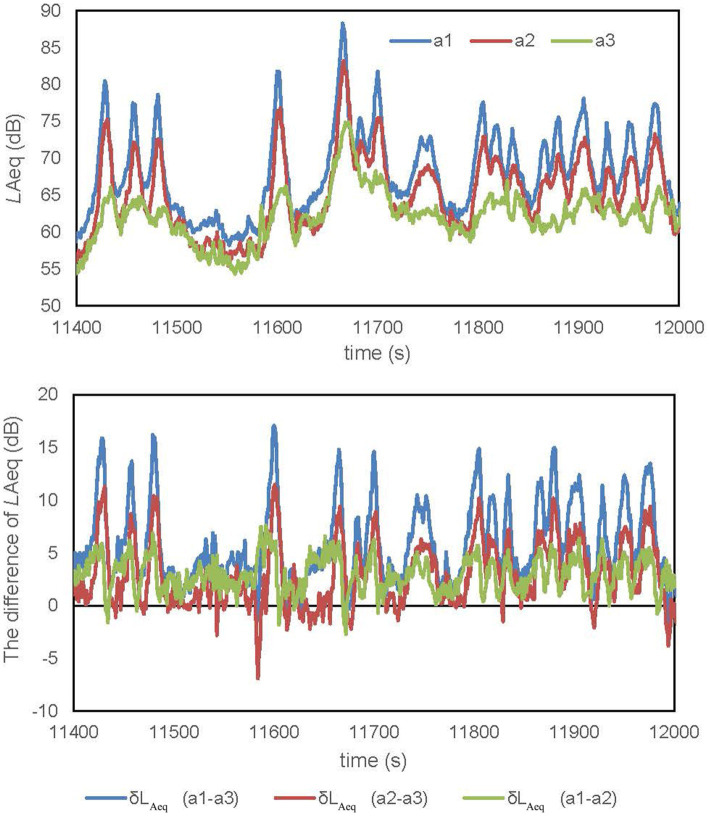
The L_Aeq_ and difference of L_Aeq_ measurement at three locations at section a (high density) of study site no. 1 (shinji Lake stroll way).

Figure 4 shows the fluctuations in sound pressure that were measured at three locations of section a in study site 1, the Shinji Lake Stroll Way, where tree density is high. The level differences occur between the three measurement locations: along the road (a-c1), behind the roadside trees (a-c2), and on the sidewalk away from the trees (a-c3). The difference in L_Aeq_ values observed at different distances from the road represents the physical noise reduction effect attributed to the presence of roadside trees. The notable level difference observed at sections a and b, characterized by high tree density, compared to section c, which has very little or no plants, highlights the potential influence of trees in attenuating noise levels. These findings are consistent with similar results obtained in other study areas, demonstrating a recurring pattern of physical noise attenuation provided by trees (Wickramathilaka et al., 2022). Accordingly, tree can effectively reduce noise levels by reflecting, diffracting, and absorbing sound waves. The amount of noise reduction depends on a variety of factors, including the type of tree, the distance between the tree and the noise source, and the frequency of the noise.

Table 3 shows the level differences calculated for the three measurement locations at three sections of all study sites. At the Shinji Lake stroll way, the average level difference due to the roadside trees and their distance between Sound Level Meter 1 and Sound Level Meter 3 is 7.7 dB (of which the attenuation calculated by the distance is 4.1 dB). It is worth noting that this level difference is noticeable to the human ear. However, the average level difference due to the distance between Sound Level Meter 2 and Sound Level Meter 3 is 4.2 dB, which is assumed to be the distance attenuation effect. The average level difference due to the roadside trees and their distance between Sound Level Meter 1 and Sound Level Meter 2 is 3.5 dB (of which the distance attenuation amount is 1.8 dB), and the average level difference due to the influence of the roadside trees is 1.7 dB. In both cases, it is difficult for the human ear to discriminate the noise level change, as the reference indicates that a 1–3 dB difference in noise level is barely noticeable to the human ear (Marshall, 2006). The level difference is more significant in Sections A and B than in Section c.

**Table 3:** The level differences at the three measurement locations of all study sites.

	Location 1–Location 3 (effect of both roadside tree and distance)	Location 2–Location 3 (effect of distance)	Location 1–Location 2 (effect of roadside tree)
**Shinji Lake stroll way**			
Section a (high density)	7.7 dB (reduction due to distance: 4.1 dB)	4.2 dB	1.7 dB
Section b (low density)	7.7 dB (reduction due to distance: 3.4 dB)	2.9 dB	2.8 dB
Section c (no or also no tree)	3.5 dB (reduction due to distance: 3.0 dB)	1.9 dB	−0.2 dB
**Sugata Parkside**			
Section a (high density)	5.6 dB (reduction due to distance: 4.1 dB)	2.4 dB	1.2 dB
Section b (low density)	2.0 dB (reduction due to distance:4.1 dB)	−1.9 dB	1.9 dB
Section c (no or also no tree)	0.9 dB (reduction due to distance:4.1 dB)	1.7 dB	1.2 dB
**Fuzoku School Kunibiki side**			
Section a (high density)	9.7 dB (reduction due to distance: 6.7 dB)	1.4 dB	2.9 dB
Section b (low density)	6.0 dB (reduction due to distance: 6.7 dB)	0.5 dB	0.1 dB
Section c (no or also no tree)	6.3 dB (reduction due to distance: 6.7 dB)	1.8 dB	−0.9 dB

Similar noise attenuation results were observed in other study sites, quantifying the effect of trees on reducing noise. In Sections a and b, the level difference was the same, indicating the combined influence of roadside trees and distance. Section c, with no roadside trees, showed a level difference similar to the distance attenuation calculated by the formula. The distance between locations 2 and 3 in each section was nearly identical. However, the level difference decreased from Section a to Section c, indicating an increase in L_Aeq_ measured by Sound Level Meter 2. The largest level difference due to roadside trees was observed in Section b, followed by Section a with a high tree density. In Section b, although the tree density was lower, the road surface vegetation likely reduced the sound volume reflected from the road.

At Sugata Parkside, the highest level difference due to roadside trees and distance was in Section a. However, in Sections b and c, the average level difference was lower than the distance attenuation calculated by the formula, possibly due to the influence of bicycle sounds in the park affecting L_Aeq_ measured by Sound Level Meter 3. The value between Sound Level Meters 2 and 3 remained constant, but it was negative only in Section b due to the significantly higher L_Aeq_ measured in that section. The level difference due to roadside trees ranged from 1.2 dB to 1.9 dB in all sections, showing no significant variation. Thus, the physical noise reduction effect of roadside trees at Sugata Parkside was deemed negligible.

On the Fuzoku School Kunibiki side, the largest level difference due to roadside trees and distance was in Section a, while Sections b and c had similar values. The level difference due to distance showed a change of approximately 1 dB across all sections as the distance between Sound Level Meters 2 and 3 remained constant. In Section b, the influence of roadside trees did not significantly reduce traffic noise. According to Meguro and Harada’s study, a perceived noise volume reduction of halving occurs with a soundproofing effect of 10 dB or more (Meguro and Harada, 2009).

### Effect on subjective evaluation

**Figure 5:**
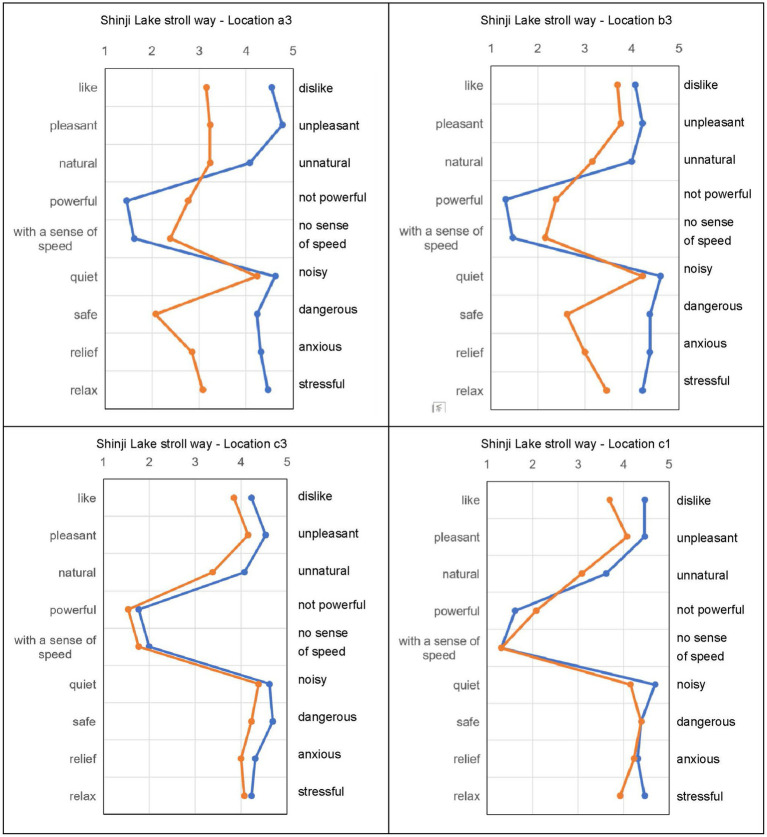
Subjective evaluation result of shinji Lake stroll way.

**Figure 6:**
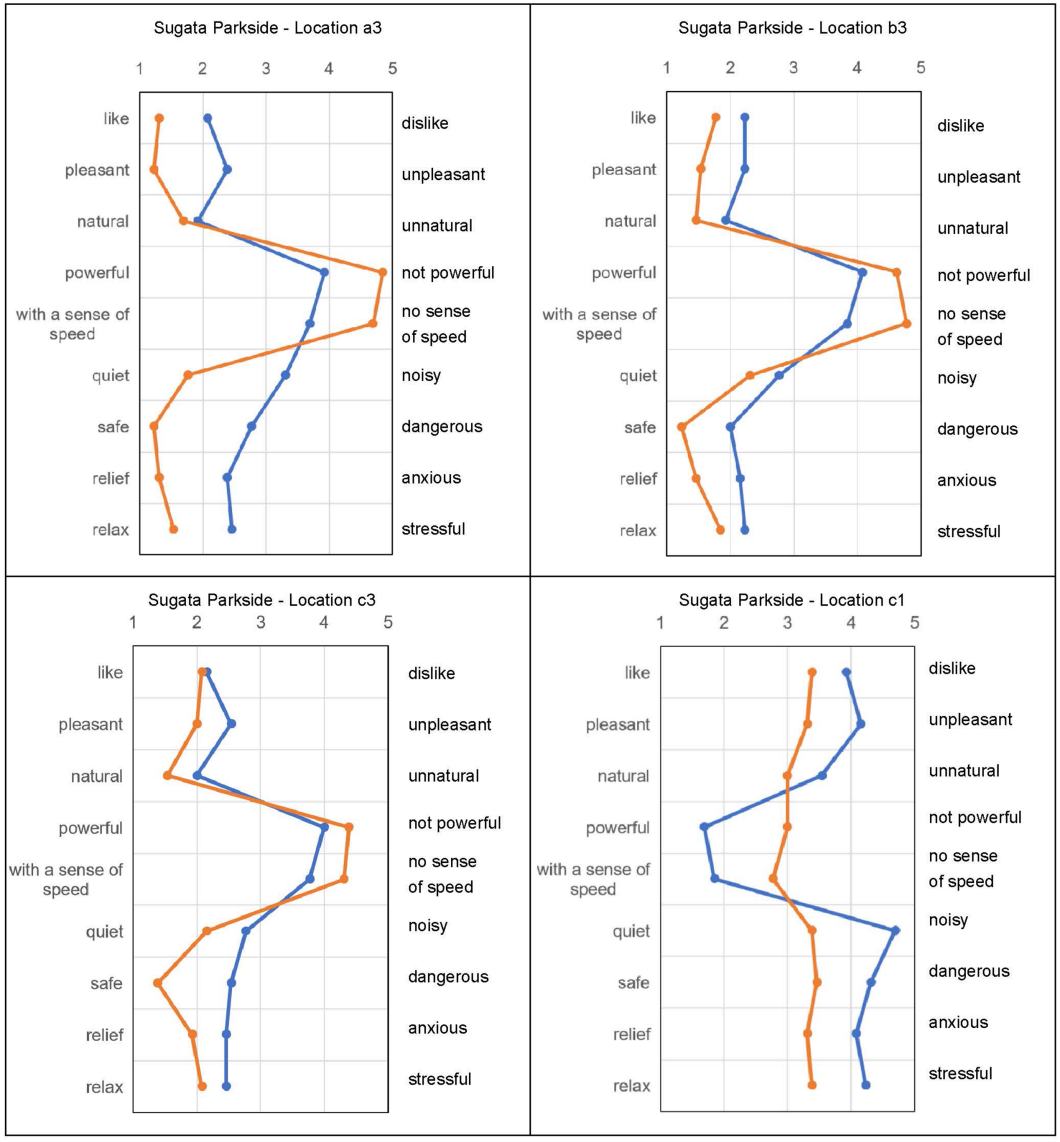
The subjective evaluation result of Sugata parkside.

**Figure 7:**
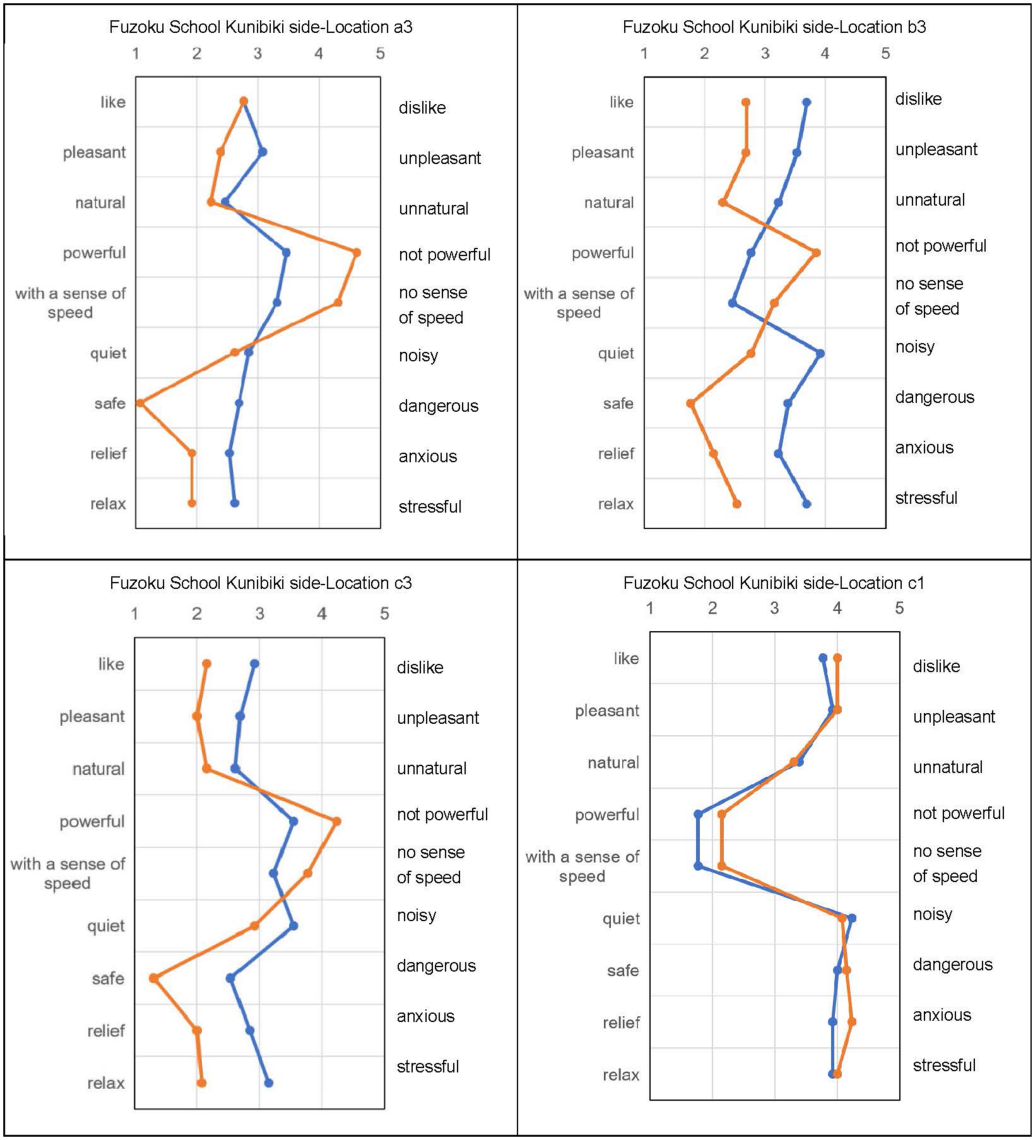
The subjective evaluation result of Fuzoku school kunibiki.

In the audio-visual evaluation experiment, participants listened to recordings in Scene 1 and then watched VR videos in Scene 2. The goal was to determine the psychological effect of green plants rather than physical noise reduction. Comparisons were made using samples recorded at Locations 1 and 3. Figures 5–7 show the subjective evaluation differences between the two scenes. At Location 1, evaluations for Scene 1 and Scene 2 were similar. However, at Locations a3, b3, and c3, the evaluations for Scene 2 were more positive. There were no significant differences in the evaluation of noise levels. Overall, Locations a3 and b3, where roadside trees were present, had more positive impressions.

In Shinji Lake Stroll Way a3, watching the video simultaneously resulted in a better impression, with significant differences in several evaluation items. Shinji Lake Stroll Way b3 showed a similar pattern, with improvements in overall impression and significant differences in certain items. Evaluations at Locations c3 and c1, where there were no trees, were negative, but the presence of roadside trees improved the overall impression. The evaluation of sound loudness showed little difference between c3 and b3.

Figure 6 demonstrates that, at high-density roadside tree locations (A3), simultaneous audio-video evaluation received more positive ratings for most items. Low-density roadside tree locations (B3) showed similar results. Locations with few roadside trees (C3) had evaluations similar to A3 and B3. Evaluations at locations without roadside trees (C1) were negative when only the sound was listened to, but became neutral when both the video and sound were experienced. Having plants, including roadside trees, in the field of vision improved the overall sound environment.

Figure 7 reveals that at high-density plant locations (A3), simultaneous audio-video evaluation improved ratings for several items, while evaluations of noise levels remained unchanged. Low-density plant locations (B3) showed improvements in overall evaluation, including noise levels. Few roadside tree locations (C3) had significant improvements in certain items, with noise levels remaining relatively unchanged. Locations without roadside trees (C1) had a consistently negative impression, with no significant difference between audio-only and audio-video evaluations. Overall, watching the video simultaneously resulted in better impressions at all three locations (a3, b3, and c3).

In summary, the visual presence of roadside trees and plants contributes to a more favorable sound environment, as reflected in subjective evaluations. However, the influence on noise reduction was not significant, indicating that the psychological effects of greenery play a larger role than physical noise attenuation.

### Different evaluations in the audio and audio-video scenes

A factor analysis with promax rotation was conducted on the entire data set, resulting in two factors for each scene. Table 4 shows the total loadings. In the audio scene, Factor 1 showed high factor loadings for attribute pairs such as: “like/dislike,” “pleasant/unpleasant,” “natural/unnatural,” “quiet/noisy,” “safe/dangerous,” “relief/anxious,” and “relax/stressful.” It accounted for 70.1% of the variance in the audio scene. Factor 2 showed high factor loadings for attribute pairs such as “powerful/not powerful” and “with a sense of speed/no sense of speed,” accounting for 54.6% of the variance in the audio scene.

**Table 4:** Rotated factor loadings in each condition.

	Audio scene		Audio-video scene	
	Factor 1	Factor 2	Factor 1	Factor 2
Like–dislike	**0.73**	−0.21	0.07	**0.86**
Pleasant–unpleasant	**0.77**	−0.17	0.21	**0.74**
Natural–unnatural	**0.67**	−0.13	0.10	**0.58**
Powerful–not powerful	−0.44	**0.53**	**−0.93**	−0.01
Sense of speed–no sense of speed	−0.02	**0.99**	**−0.89**	0.03
Quiet–noisy	**0.77**	−0.06	**0.67**	0.10
Safe–dangerous	**0.64**	−0.30	**0.63**	0.27
Relief–anxious	**0.82**	−0.13	**0.58**	0.33
Relaxed–stressful	**0.86**	−0.07	0.38	**0.57**
Suitable–unsuitable			−0.02	**0.38**

The values in bold indicate relationships with *p*-values < 0.05 or significant differences.

In the audio-video scene, Factor 1 showed high factor loadings for attribute pairs like: “powerful/not powerful,” “with a sense of speed/no sense of speed,” “quiet/noisy,” “safe/dangerous,” and “relief/anxious.” It accounted for 55.2% of the variance in the audio-video scene. Factor 2 showed high factor loadings for attribute pairs such as: “like/dislike,” “pleasant/unpleasant,” “natural/unnatural,” “relax/stressful,” and “suitable/unsuitable.” It accounted for 51.6% of the variance in the audio-video scene.

**Figure 8:**
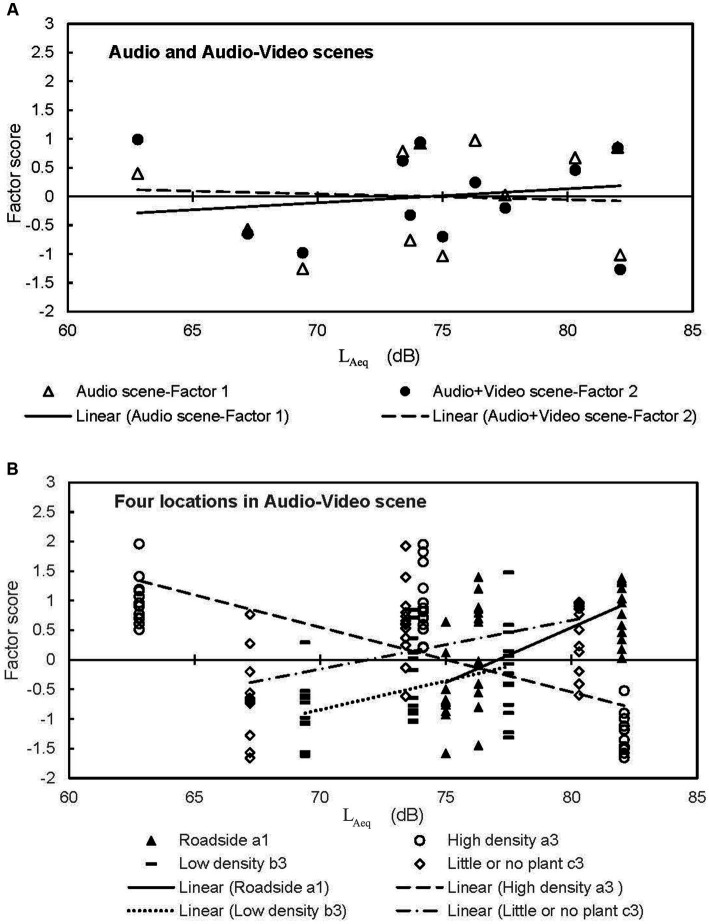
Scatterplots of the factors score vs. L_Aeq_ with regression lines **(A)** experiment scene and **(B)** locations in Audio-Video scene.

For the Audio scene, Factor 1 can be interpreted as indicating sound preference and Factor 2 can be interpreted as indicating a sense of traffic movement. For the Audio-video scene, Factor 1 can be interpreted as indicating the Physical aspect, and Factor 2 can be interpreted as indicating the emotional aspect. The linear regressions of L_Aeq_ against the score of Factor 1 with an audio scene and Factor 2 with an audio-video scene which contains the impression of sound preference and emotion, were examined. Figure 8 shows scatterplots of L_Aeq_ vs. the average factor score with regression lines according to each experiment scene. In the context of using only audio stimuli, the line slopes upward progressively as the noise level increases. However, in the context of audio and video, the rating line tends to slope downward as the sound pressure level increases. In other words, the test participants’ evaluations were more positive when watching the video and listening to the audio.

### Relationship between physical and psychological effects

Physical index including sound pressure level (dB), loudness (sone), and sharpness (acum) were calculated from the samples of the binaural recording. As shown in Tables 5, 6, the correlation between the subjective evaluation and the physical index was calculated using the correlation coefficient. The values in bold indicate relationships with *p*-values < 0.05 or significant differences.

**Table 5:** The correlation coefficient between subjective evaluation and physical index.

	L_Aeq_ (dB)	Loudness (sone)	Sharpness (acum)
**Audio stimuli**			
Like	0.107	0.273	**0.351**
Pleasant	**0.152**	**0.272**	**0.348**
Natural	0.058	0.146	0.275
Powerful	−0.082	−0.239	**−0.444**
With a sense of speed	−0.099	−0.290	**−0.448**
Quiet	**0.166**	**0.306**	**0.337**
Safe	0.191	0.287	**0.327**
Relief	0.098	0.236	**0.367**
Relax	**0.125**	0.296	**0.344**
**Audio-visual stimuli**			
Like	−0.085	0.085	**0.305**
Pleasant	**−0.028**	**0.146**	**0.337**
Natural	−0.013	0.088	0.233
Powerful	0.028	−0.127	**−0.375**
with a sense of speed	−0.055	−0.241	**−0.361**
Quiet	**−0.085**	0.045	**0.326**
Safe	0.028	0.278	**0.376**
Relief	−0.047	0.197	**0.356**
Relax	**−0.035**	0.159	**0.365**
Suitable	−0.001	0.025	0.114

**Table 6:** The correlation coefficient between subjective evaluation and physical index for three sections of roadside density in A + V experiments.

	Section a			Section b			Section c		
	L_Aeq_ (dB)	Loudness (sone)	Sharpness (acum)	L_Aeq_ (dB)	Loudness (sone)	Sharpness (acum)	L_Aeq_ (dB)	Loudness (sone)	Sharpness (acum)
Like	**−0.421**	−0.110	**0.369**	**0.384**	0.254	**−0.361**	**0.446**	0.226	**0.588**
Pleasant	**−0.349**	−0.045	**0.330**	**0.456**	**0.374**	−0.263	**0.471**	0.213	**0.671**
Natural	**−0.227**	−0.073	**0.269**	**0.338**	0.251	−0.258	**0.329**	0.178	**0.410**
Powerful	**0.317**	0.084	**−0.259**	**−0.370**	**−0.432**	−0.082	**−0.403**	−0.150	**−0.635**
Sense of speed	0.187	−0.109	**−0.335**	**−0.610**	**−0.628**	0.058	**−0.317**	−0.099	**−0.538**
Quiet	**−0.284**	−0.042	**0.359**	0.175	0.149	−0.089	0.144	−0.055	**0.439**
Safe	**−0.322**	−0.002	**0.266**	0.305	**0.399**	0.167	**0.776**	**0.648**	**0.523**
Relief	**−0.369**	−0.022	**0.360**	**0.355**	0.302	−0.179	**0.504**	**0.381**	**0.418**
Relaxed	**−0.362**	−0.074	**0.310**	0.299	**0.334**	0.030	**0.498**	0.302	**0.559**
Suitable	−0.096	−0.028	0.168	0.290	0.258	−0.121	0.050	−0.072	0.257

The values in bold indicate significant correlations.

The magnitude of the absolute value of the correlation coefficient roughly corresponds to four levels of correlation: 0 to 0.2 represents no correlation; 0.2 to 0.4 represents weak correlation; 0.4 to 0.7 represents moderate correlation; and 0.7 to 1 represents strong correlation. The results show a weak correlation between the subjective evaluation and the physical index, with values ranging from 0.2 to 0.4. Among the physical indexes, sharpness was found to have a stronger correlation with subjective evaluation than the other physical indexes. A higher value of sharpness was found to be associated with a negative evaluation of sound and is generally used to interpret individual sound characteristics.

### Contribution of plant density

While vegetation can have some impact on reducing traffic noise levels, the effectiveness depends on various factors, such as the type of vegetation and its density (Ow and Ghosh, 2017). In this section, the role of trees density and the visual environment in enhancing the positive perception of sound was examined. The relationship between sound levels and the subjective evaluation represented by the score of Factor 2 was established for four groups: (a1) standing right on the roadside, (a3) high density trees, (b3) low density trees, and (c3) almost no trees. As seen in Figure 8, the group with a high density of trees had a significant reduction in negative evaluation, as shown by the sharp downward slope in the graph. In contrast, the other groups all showed an upward slope, indicating that the positive effect was not found without the presence of high-density trees. However, among these groups, the roadside view (where trees and vehicles were seen at the same time) and the low-density tree group still showed a lower negative evaluation compared to the group with almost no trees.

Multiple regression analysis was performed to clarify the contribution degree of tree density (1, high density, 2: medium or low density, 3: little vegetation, 4: along the road) and noise level (as a continuous variable) as independent factors to the subjective auditory and visual evaluation. A dummy variable for the experiment stimuli (1, audio, 0: audio-video) was included in the regression model as an independent factor to determine whether the experiment scenes or the presence of roadside trees affected the evaluation.”

Table 7 shows the results of the analysis for the evaluation attribute pair “quiet-noisy” (1: very quiet, 2: slightly quiet, 3: neither quiet nor noisy, 4: slightly noisy, 5: very noisy). The evaluation was found to differ significantly between step 3 (little vegetation) and step 2 (medium density), meaning that little vegetation was perceived as noisier than medium density. However, the evaluation of step 2 (medium density) and step 1 (high density), as well as step 4 (roadside) and step 3 (little vegetation), showed no significant difference and a negative coefficient estimate. This result may reflect a sensitivity to sound intensity that can vary with the range of tree density. Specifically, the evaluation of the interaction was found to be most strongly correlated with the difference between the medium and little levels of tree density.

**Table 7:** Multiple regression relationship between the L_Aeq_ (dB), plant density, experiment scene, and the evaluation of item “quiet-noisy” and “safe-dangerous.”

Evaluation item	Term	Estimate	Std error	t ratio	Prob>|t|
Quiet-noisy	Intercept	3.851	2.408	1.599	0.127
	L_Aeq_ (dB)	−0.002	0.033	−0.050	0.960
	Audio/A + V[0]	0.317	0.172	1.841	0.082
	Density[2–1]	−0.858	0.488	−1.759	0.096
	Density[3–2]	1.026	0.488	2.104	<0.05
	Density[4–3]	−0.121	0.506	−0.240	0.813
Safe-dangerous	Intercept	2.073	3.063	0.677	0.507
	L_Aeq_ (dB)	0.020	0.042	0.475	0.640
	Audio/A + V[0]	0.542	0.219	2.471	<0.05
	Density[2–1]	−1.498	0.620	−2.414	<0.05
	Density[3–2]	1.075	0.620	1.734	0.100
	Density[4–3]	−0.017	0.643	−0.027	0.979

The results of the analysis performed on the “safe-dangerous” evaluation (1: very safe, 2: slightly safe, 3: neither safe nor dangerous, 4: slightly dangerous, 5: very dangerous) shows that the evaluation differed significantly between audio and audio-video stimuli, with audio stimuli being evaluated as more dangerous. On the other hand, there was a significant difference between (2) medium density and (1) high density, with (2) medium density being evaluated as safer. The positive estimate for the experiment stimuli variable and the negative estimate for the density variable indicate these relationships. There was no significant difference found in the other variables. The apparent discrepancy in the evaluation of safety and the density of trees may be due to the fact that pedestrians feel safer when they can see a portion of the road, rather than when the trees completely obscure their view.

## Discussion

### Soundscape and landscape coexistence

This study clarifies the extent to the vision of roadside trees, planted at different densities, influences pedestrians’ perception of traffic noise. The visual landscape significantly affects preferences for the sound environment. The study emphasizes the importance of understanding the subjective experience of sound, or “soundscape,” in urban environments, rather than relying solely on objective physical descriptions. Our study support previous research on the association between visual landscapes and the sound environment, highlighting how they can influence people’s perception and preferences. This align with studies that consider individual differences in perception and sensitivity to sound, as well as the role of context and meaning in shaping our reactions to noise (Field, 1993; Job, 1999). These findings underscore the significance of adopting a multidisciplinary and human-centered approach to comprehending sound within our environment.

The influence of plants on perceptions of quietness and safety was examined through a comparison of responses to samplings with varying plant density. This finding confirms the positive effects of plants and greenery on perceived quietness and safety, particularly in urban environments. It is consistent with the results of a previous web-based experiment that assessed restoration likelihood among Icelandic adults. In that study, participants who viewed urban residential streetscapes with a higher number of trees reported feeling more restored (Pall, 2015). This agreement suggests that perceived quietness and safety and restoration is correlation related and may mutually enrich each other.

### Role of vegetation planned as noise barriers

The noise level ranges measured at most exposed sections of the three pedestrial route in Matsue City ranged from 55.0 to 73.8 dB, indicating the uncomfortable condition of the outdoor environment regarding acoustic quality. The World Health Organization (WHO) recommends that outdoor noise levels should not exceed 55 decibels (dB) during the day and 45 dB at night to protect public health (World Health Organization, 2011). However, the WHO notes that traffic noise levels can range from 50 to 85 dB or higher, depending on the location and traffic conditions. This study determined the modest effects of plants on reducing such high traffic noise levels. This is associated with studies suggest that while plants can have a beneficial effect on noise reduction, their effect may be limited and should not be relied upon as the only means of reducing noise. Other factors, such as the placement and design of barriers, may also need to be considered. Vegetation was proved to be able to act as a barrier to noise, absorbing sound waves and reducing their transmission (Van Renterghem et al., 2012). The study in Flanders, Belgium, suggests that creating and preserving quiet areas and routes, as well as incorporating vegetation, can be an effective way to mitigate the negative impact of noise pollution on human health and well-being (Renterghem and Botteldooren, 2011). Roadside trees can be more impactful on reducing noise levels, with the larger, denser vegetation providing greater noise reduction. The present study suggests that increasing the density of vegetation in urban design can be an effective way to mitigate noise pollution.

It was determined that, despite having no significant noise reduction effect physically, the vision of roadside tree effectively creates a soundscape that improves the psychological health of urban residents. In other words, it suggest that exposure to greenery, such as roadside trees, can have a positive impact on psychological health. Gladwell et al. found that exposure to views of nature has a positive effect on autonomic control, which can reduce stress and improve health (Gladwell et al., 2012). Our study provides an evidence on the positive effects of vision of roadside trees on reducing stress due to traffic noise.

### The arrangement of roadside trees and walking spaces in public spaces

It is important to carefully consider the placement of roadside trees in public spaces near traffic due to their significant impact on pedestrians’ perception and appreciation. The findings of the present study contribute to our understanding of the best placement of roadside trees in public spaces near traffic. Vegetation has been proven to reduce traffic noise in urban areas, with the extent of noise reduction depending on factors such as the distance between the noise sources and the trees, the height and density of the trees, and the frequency of the noise (Ow and Ghosh, 2017; Margaritis et al., 2018). Investigations on the effect of roadside vegetation and sound barriers on the propagation of traffic noise found that vegetation had a greater impact on reducing noise at high frequencies, while sound barriers are more effective in reducing noise at low frequencies (Kalansuriya et al., 2009; Van Renterghem et al., 2012). Our study suggests that effective psychological noise reduction can be enhanced by considering factors such as the appropriate length and depth of street trees, their distance from the noise source, and the planting plan. In section c of this study, we found that tree fences at low density can still effectively obstruct noise when combined with shrubs and grass. This conclusion is consistent with the findings of a study which examined the effectiveness of different types of greenery in reducing noise in Delhi (Tyagi et al., 2006). The effectiveness of noise reduction varied depending on the frequency of the noise and the height and density of the greenery. This study suggest that the placement of roadside trees should be carefully considered, taking into account factors such as the distance from the noise source, the length and depth of the trees, and the layout of the planting plan.

### Study limitations and further study

One limitation of the study is the modest sample size and reliance on self-reported subjective evaluations, which may be influenced by individual biases and preferences, potentially affecting the research findings. To enhance the study’s robustness, the inclusion of physiological responses alongside subjective evaluations would provide a more comprehensive assessment of the sound environment. Furthermore, while the study examines the effects of different densities of roadside trees on perceptions of quietness and safety. However, it does not explicitly addressed other important variables, such as tree species, height, and maintenance practices. Future research should consider investigating these variables to provide more nuanced insights. In further study, in addition to the laboratory-based subjective evaluation method using simulation samples, an on-site assessment method, such as the soundwalk method, could be considered.

## Conclusion

Roadside trees have a positive psychological impact on urban residents by enhancing the soundscape and reducing stress caused by traffic noise. The visual presence of roadside trees at different densities influences pedestrians’ perception of traffic noise and contributes to their overall perception of the sound environment. Careful consideration should be given to the placement of roadside trees, taking into account factors such as the distance from the noise source, the size and arrangement of the trees, and the overall planting plan. Integrating green infrastructure, such as roadside trees, into urban design can contribute to the creation of more pleasant and healthier environments for urban residents.”

## Data availability statement

The raw data supporting the conclusions of this article will be made available by the authors, without undue reservation.

## Ethics statement

The studies involving human participants were reviewed and approved by Fukushima University. The patients/participants provided their written informed consent to participate in this study.

## Author contributions

TN: conceptualization, methodology, writing—original draft, and funding acquisition. MM: data curation, review, and editing. All authors contributed to the article and approved the submitted version.

## Funding

This work was supported by the Shimane University‘s Diversity Promotion Program (2021-B Project).

## Conflict of interest

The authors declare that the research was conducted in the absence of any commercial or financial relationships that could be construed as a potential conflict of interest.

## Publisher’s note

All claims expressed in this article are solely those of the authors and do not necessarily represent those of their affiliated organizations, or those of the publisher, the editors and the reviewers. Any product that may be evaluated in this article, or claim that may be made by its manufacturer, is not guaranteed or endorsed by the publisher.
